# Three-dimensional structure of the orbicularis retaining ligament: an anatomical study using micro-computed tomography

**DOI:** 10.1038/s41598-018-35425-0

**Published:** 2018-11-19

**Authors:** Jehoon O, Hyun-Jin Kwon, You-Jin Choi, Tae-Hyeon Cho, Hun-Mu Yang

**Affiliations:** Department of Anatomy, Yonsei University College of Medicine, Seoul, Republic of Korea

## Abstract

The orbicularis retaining ligament (ORL) is an important structure for maintaining the eyelid and cheek skin and contouring the characteristic facial appearance. However, the ORL is a delicate structure that is easily damaged in manual dissection. This study aimed to comprehensively investigate the ORL using a micro-computed tomography (mCT) with phosphotungstic acid (PTA) preparation for the acquisition of its three-dimensional information non-destructively. Twenty-two specimens were obtained from non-embalmed human cadaver (mean age 73.7 years). Multidirectional images of the mCT showed that the ORL consisted of continuous tiny plates with a multilayered plexiform shape. The modified Verhoeff Van Gieson staining and immunofluorescence revealed a ligamentous tissue consisting of multiple fibroelastic bundles. The preorbicularis fibres of the ORL had more layers and a more intricate arrangement than its retro-orbicularis fibres. The number, complexity and ambiguity of the ORL fibres increased in the lateral area and their density and extent increased near the dermis. Its dermal anchorage was shown as a confluence of its fibroelastic tissue into the dermis. The ORL comprises a multilayered meshwork of very thin continuous fibroelastic plates and its related cutaneous deformities might be a complicated outcome of subcutaneous tissue shrinkage, lipid accumulation and ORL retention.

## Introduction

The midcheek skeleton and overlying soft tissue delicately maintain the mobility and stability of the midface. The orbicularis retaining ligament (ORL) is a particularly important structure for maintaining the eyelid and cheek skin and contouring the characteristic facial appearance. The ORL is an osseocutaneous septum-like structure originating from the periosteum near the orbital septum and encircling the lower orbital rim^1,2^. This ligament is also known as the orbitomalar ligament, tear trough–orbicularis retaining ligament complex and malar septum^3–6^. It reportedly has a muscular insertion into the deep surface of the orbicularis oculi muscle (OOc), and a cutaneous insertion along the malar and nasojugal fold^2,3^. Although the detailed delineation of its attachment has varied among previous studies, it is clear that the ORL is involved in the production of cutaneous grooves at the eyelid–cheek junction. Wong *et al*.^7^ described a distinct groove consisting of the nasojugal groove (tear trough) medially and the palpebromalar groove laterally, and Schiller^1^ reported a continuous orbitomalar sulcus overlying the ORL. In addition, Hashem *et al*.^6^ described a second cheek bulge, the malar mound or festoon that appeared below a palpebromalar groove.

The exact pathophysiology of cutaneous deformities is still debated. It is certain that aging changes the ORL morphology and the distribution of facial fat. As an osteocutaneous ligament, the ORL compartmentalizes a midcheek subcutaneous space in the preseptal space and the prezygomatic-premaxillary space^6,8^. The shrinkage and drooping of demarcated fat pads produced by the ORL deepens the cutaneous groove^9^. Muscular or cutaneous anchorage of the ORL also changes the ligamentous elasticity and tension with aging. A laxity of the ligament enables the orbital fat to drop, and it additionally forms a preseptal protrusion with a weakened orbital septum^3^. These changes together result in an unfavourable appearance with a bulging lower eyelid or a sagging malar bag. An understanding of the detailed anatomy of the ORL is therefore very important for midfacial rejuvenation and lower blepharoplasty^1,4^.

Detailed anatomical information is not yet available about the ORL regarding its pattern of layering, periosteal or cutaneous anchorage, relationship with muscle fibres, arrangement of ligament fibres and the histological composition of fibres. There have been numerous clinical and anatomical studies of the ORL, which have simply shown this ligament to be a thin septal sheath during clinical procedures or after manual dissections^10–12^. However, histological studies indicate that the ORL is actually a delicate and vulnerable structure that is easily damaged in manual manipulations. Moreover, it is very difficult to observe a structure such as the ORL stereoscopically in a simple histological examination. Verifying the previously reported ORL anatomy and revealing its detailed intact morphology require a fine radiological methodology as an accurate non-destructive observational technique.

The aim of this study was to comprehensively investigate the ORL using a method that overcomes the previous observational limitations. To this end, micro-computed tomography (mCT) was combined with phosphotungstic acid (PTA) preparation for enhancing the contrast of soft tissue^13–17^. This approach allowed the acquisition of three-dimensional (3D) information of the ORL non-destructively, and this information was additionally verified using modified Verhoeff Van Gieson staining (VG) and immunofluorescence (IF).

## Results

**Figure 1:**
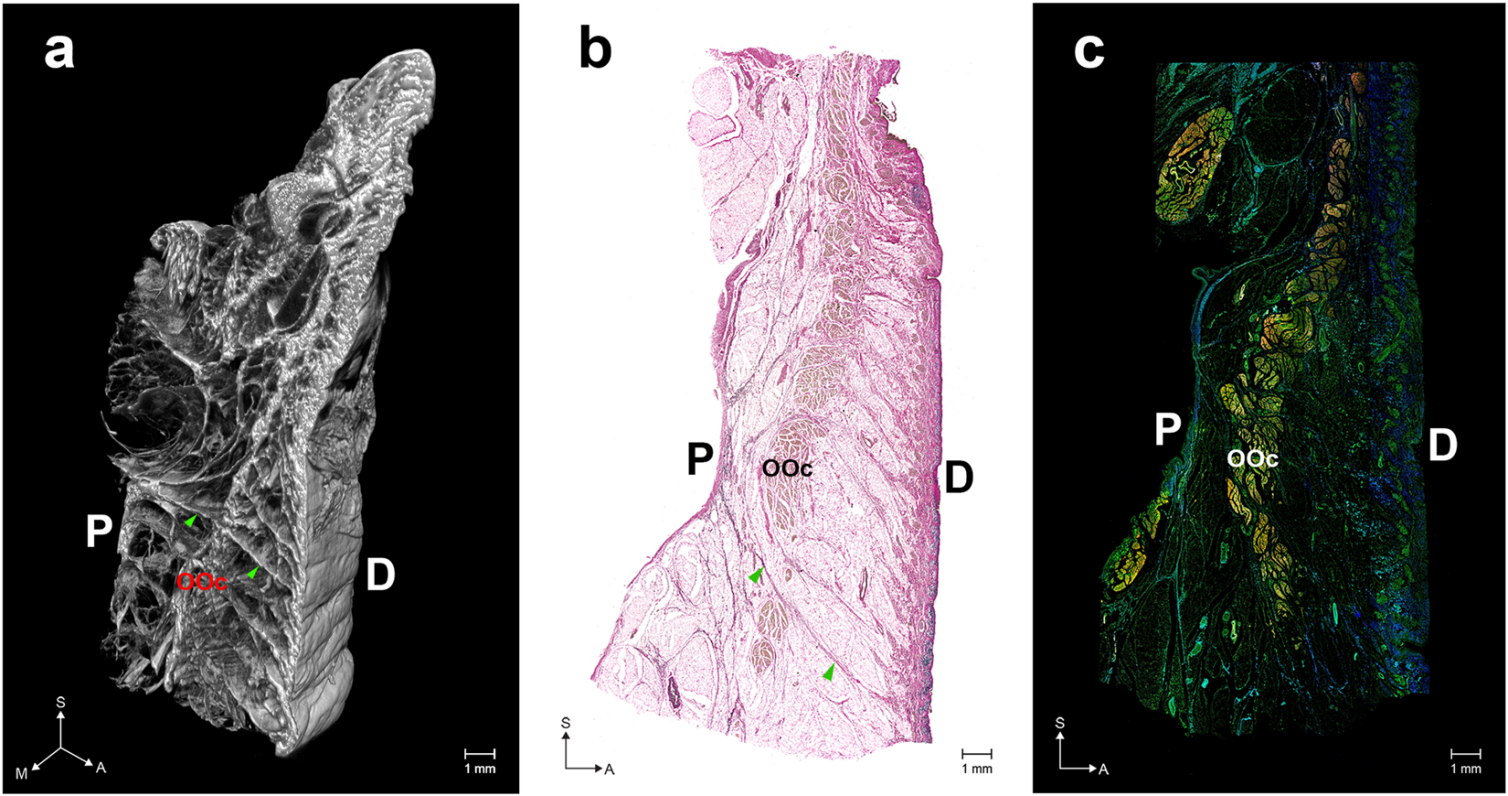
Overall structure of the orbicularis retaining ligament (ORL). (**a**) Three-dimensional (3D) morphology reconstructed from micro-computed tomography (mCT) image sections. (**b**) Modified Verhoeff Van Gieson staining (VG) image. (**c**) A merged immunofluorescence (IF) image (elastin, blue; collagen type I, green; actin, red). Arrowheads indicate a direct fibre from the periosteum (P) to the dermis (D). OOc, orbicularis oculi muscle. S, sagittal; M, medial; A, anterior. The 3D morphology of the ORL is also shown as a movie in Video S1. VG images of the medial, central and lateral portions of the ORL are shown in Fig. S1. Three individual IF images of elastin, collagen type I and actin are separately presented in Fig. S2.

The ORL typically appeared as a distinct retaining ligament below the orbital rim (Fig. 1). The mCT images showed that the ORL consisted of oriented ligamentous tissue between a periosteum and dermis (Fig. 1a). The ligaments descended in an anterior direction and continuously extended in a horizontal direction (Supplementary Video S1). The histological analyses performed using VG and IF revealed a ligamentous tissue consisting of multiple fibroelastic bundles along the overall area of the lower orbicular region (Fig. 1b,c and Supplementary Fig. S1).

### Retro-orbicularis and preorbicularis fibres of the ORL

The continuous bundles of the ORL could be divided into retro-orbicularis and preorbicularis fibres. The retro-orbicularis fibres consisted of a single or double bundle between the periosteum and the OOc. Arborization of the retro-orbicularis fibres was evident within the muscle. Ramified fibres were continuous with the preorbicularis fibres ahead of the muscle. However, there were a few bundles of the ORL that lacked the intramuscular ramification and seemed to form a single continuous fibre from the periosteum to the dermis (arrowheads in Fig. 1a,b).

### Multilayered structure of the ORL

**Figure 2:**
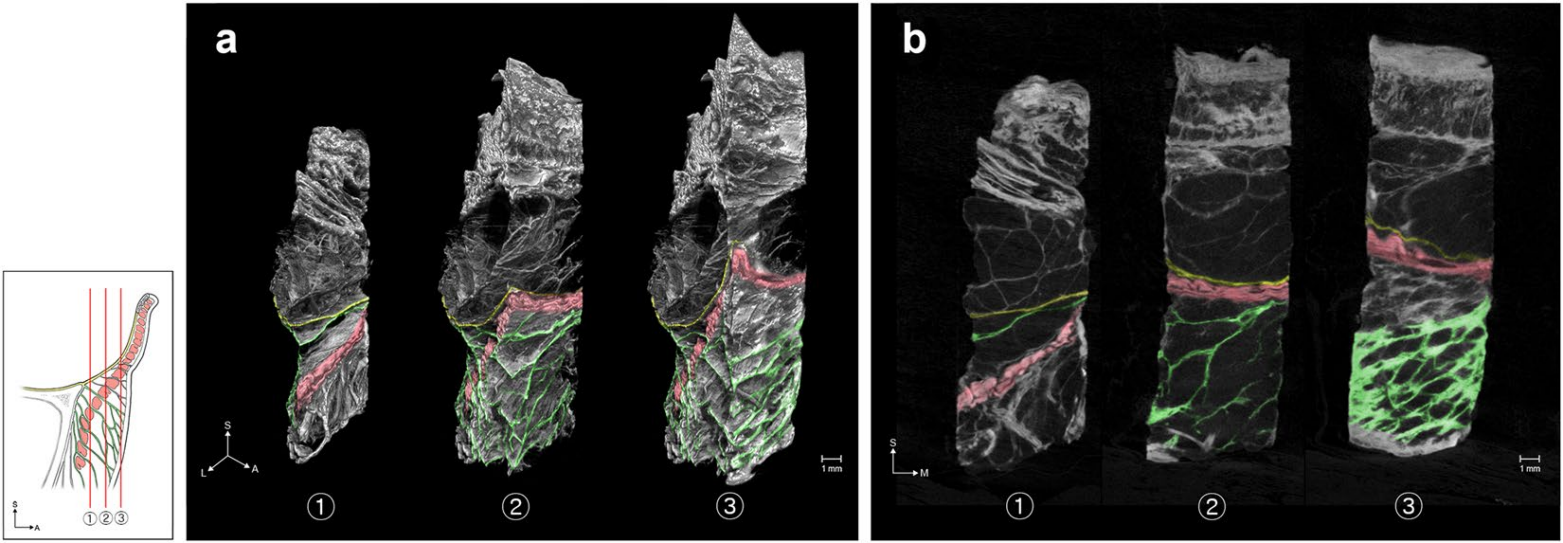
The coronally sectioned 3D morphology (**a**) of the ORL in mCT and the corresponding sectional images (**b**). The sectional planes are located posterior (1) and anterior (2) to the OOc, while (3) is the most-anterior section near to the skin. Green, yellow and red indicate the ORL, the orbital septum and the section of the OOc, respectively. L, lateral.

**Figure 3:**
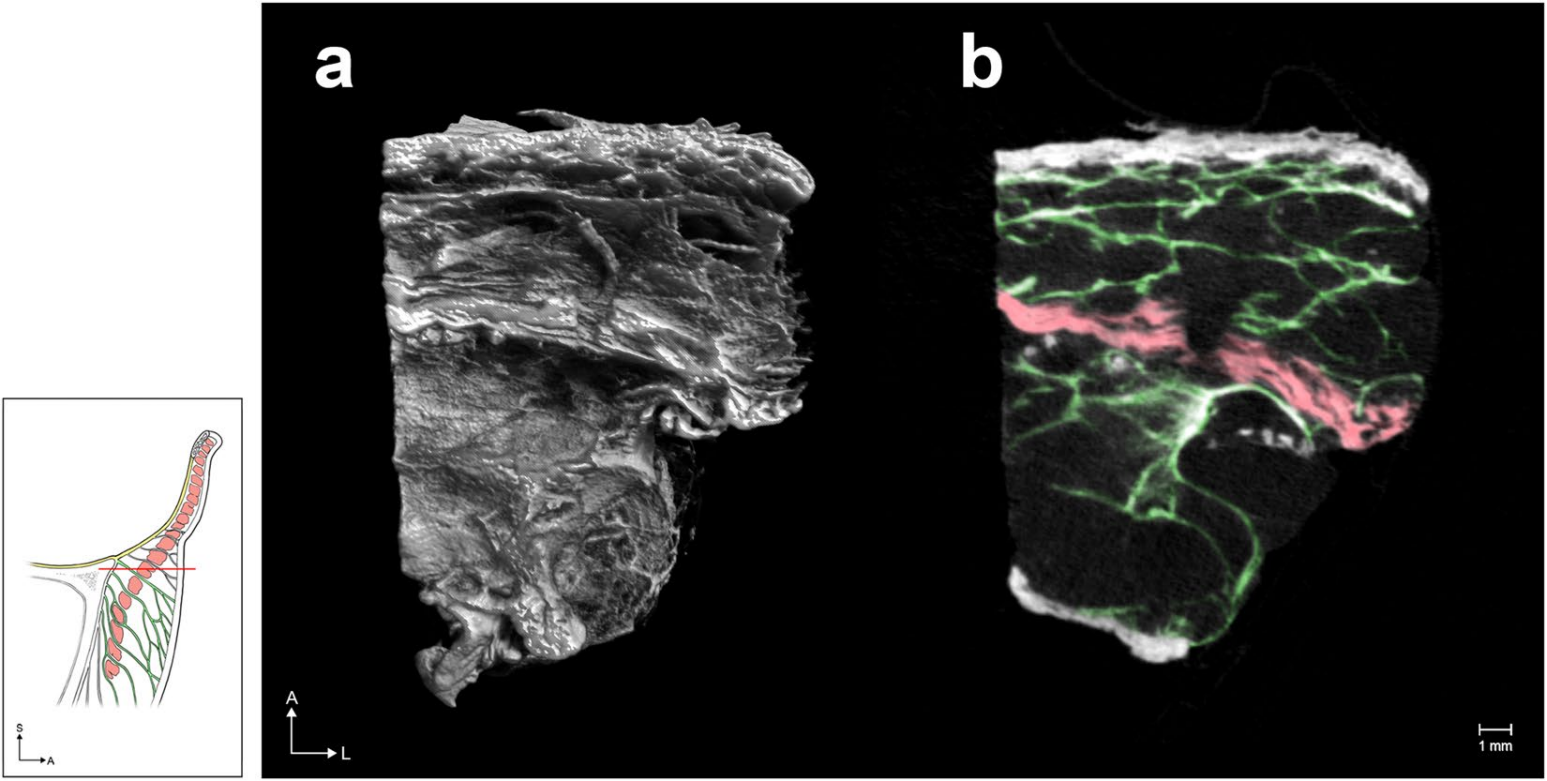
The horizontally sectioned 3D morphology (**a**) of the ORL in mCT and the corresponding sectional image (**b**). Green and red indicate the ORL and the section of the OOc, respectively.

**Figure 4:**
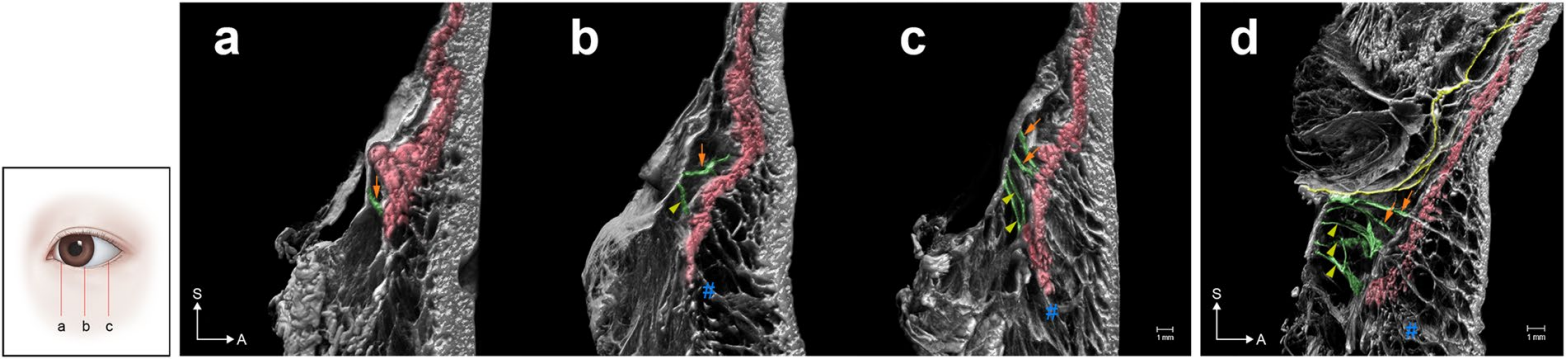
Sagittal sections of the 3D morphology of the ORL reconstructed from mCT images. The sectional planes were located at the medial (**a**), central (**b**) and lateral (**c**) portions of an ORL specimen. The retro-orbicularis fibres of the ORL (green) pierced the OOc (OOc, red). The upper fibres (arrow) pierced the palpebral and orbital portions of the OOc. The lower fibres (arrowheads) pierced the orbital portion of the OOc. The lowermost preorbicularis fibres (#) attached to the prezygomatic skin are shown in (**b**), (**c**) and (**d**). (**d**) Sagittal section of the lateral portion of another ORL specimen showing the uppermost fibre originating from the orbital septum (yellow).

Multidirectionally sectioned images obtained using mCT (Figs 2–4) showed that the ORL consisted of continuous tiny plates with a multilayered plexiform shape. In coronal sections (Fig. 2), the structure of the retro-orbicularis fibres simply comprised one to five layered plates, whereas the preorbicularis fibres had more layers and a more intricate arrangement. The density and extent of their fibroelastic tissue increased near the dermis. In horizontal sections (Fig. 3), the ORL appeared as a filamentous meshwork with arborization. Given that the layered plates of the ORL were oriented anteroinferiorly, this arborized filamentous structure appeared obliquely in sections of its multilayered structure. The borders of the ORL were mostly identified in sagittal sections (Fig. 4), and its upper border tended to be clearer than the lower one.

### Regional anatomy of the ORL

The number, complexity and ambiguity of the fibres comprising the ORL increased in the lateral area (Fig. 4). The number of retro-orbicularis fibres increased laterally. Although the ability to definitively count the number of preorbicularis fibres was impeded by its structural complexity, there was a clear trend for the number of fibres to increase laterally.

The palpebral portion of the OOc was defined as a preseptal part closely attached to the dermis with a thin subcutaneous layer. Its orbital portion was defined as a lower part behind a thick subcutaneous adipose layer. The lowermost position of the palpebral portion was located slightly above the level of the orbital rim. The uppermost retro-orbicularis fibres mainly pierced the OOc at this level. Other fibres broadly pierced the orbital portion. In particular, some fibres attached to the prezygomatic skin after piercing a lower part of the orbital portion of the OOc. The upper retro-orbicularis fibres mostly originated at a periosteum overlying the orbital rim, while some fibres originated between the periosteum and the orbital septum (Fig. 4a–c). However, the ORL did not originate from the middle or anterior part of the orbital septum (Fig. 4d). A dermal attachment of the ORL descended slightly from the medial to the central area, and the ORL dispersed ambiguously in the lateral area.

### Relationship with the muscle and dermis: detailed histological findings

**Figure 5:**
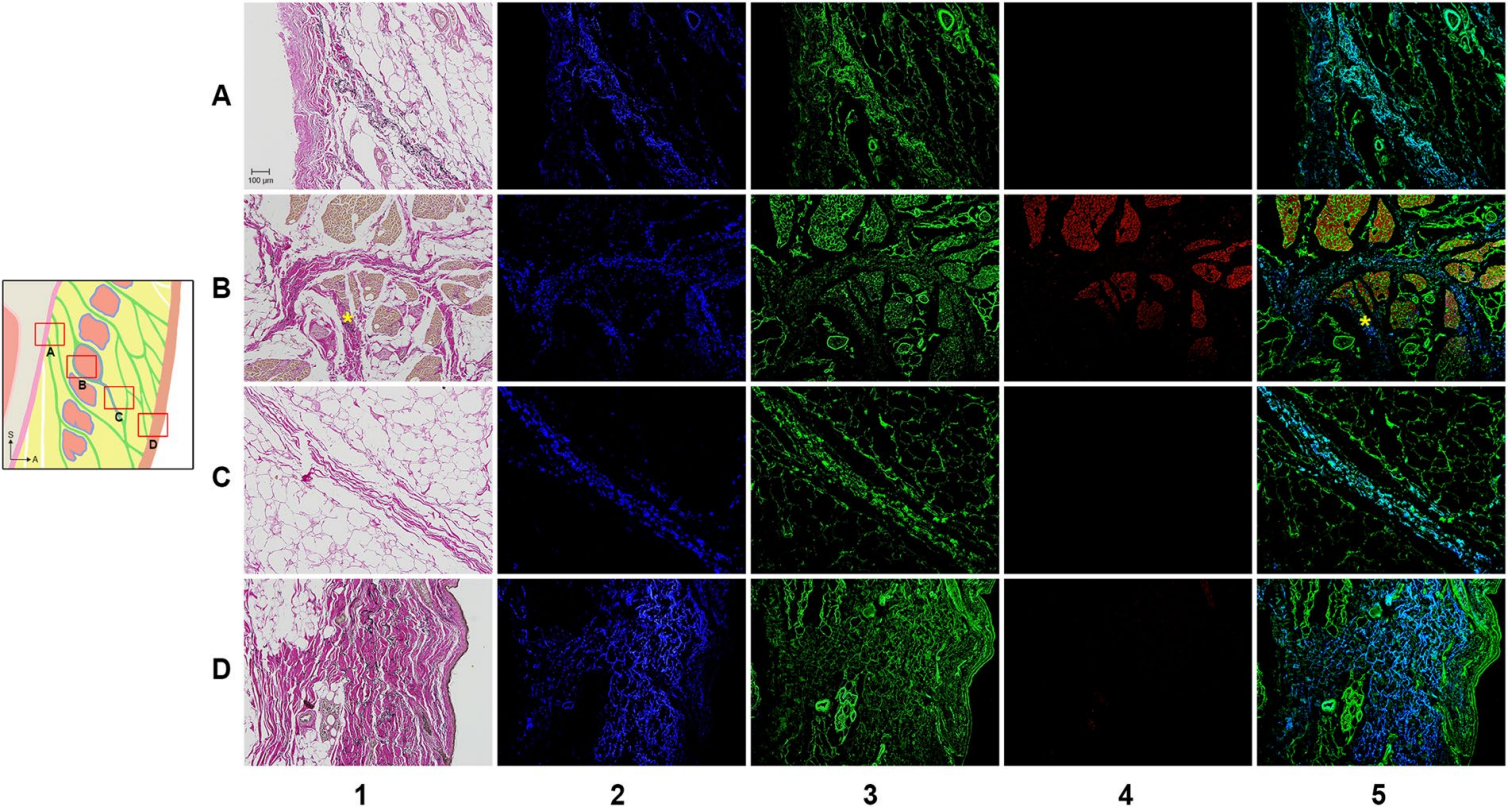
VG and IF images of the ORL. The periosteal (row A), intramuscular (row B), preorbicularis (row C) and dermal (row D) regions of the ORL were observed by VG (column 1) and IF (columns 2–5). There were immunopositive reactions for elastin (column 2, blue), collagen type I (column 3, green) and actin (column 4, red). The column 5 shows a merged image of the images in columns 2–4 image. Asterisks indicate confluence of the perimysium into the ORL fibres.

The following four regions of the ORL were examined histologically in detail: the periosteal, intramuscular, preorbicularis and dermal regions (Fig. 5). The detailed histological findings were almost entirely consistent with the morphological features evident in the mCT images. The periosteal elastin was continuous with that in the ligament and the dermis, while elastin was virtually absent from the perimysium. Type I collagen was found in the periosteum, perimysium, dermis and ORL. The muscle fibre did not join the ligament nor directly anchor to the dermis (column 4 in Fig. 5 and Supplementary Fig. S2). Even in the medial area, there was no muscle fibre below the bundles of the ORL even though the bundles were located very close to the palpebral portion of the OOc.

**Figure 6:**
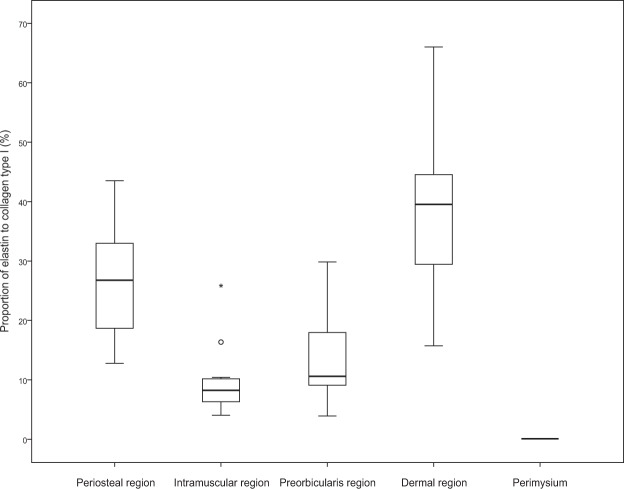
Regional immunoactivity ratio of elastin to collagen type I in the four regions of the ORL presented in Fig. 5. All regions of the ORL contained elastin; the proportions were 25.6 ± 9.4% (mean ± SD), 9.8 ± 6.3%, 13.9 ± 8.7% and 38.1 ± 14.3% in the periosteal, intramuscular, preorbicularis and dermal regions, respectively. In contrast, there was no elastin in the perimysium. Each box plot shows the median, 25th and 75th percentiles, and range. The circle and asterisk indicate outliers.

Like the mCT findings, the histological findings also presented a distinct intramuscular ramification of the retro-orbicularis fibres (row B in Fig. 5). Their fibroelastic tissue was usually separated from the perimysium. Elastin-positive ligamentous tissue of the retro-orbicularis fibres could be distinguished from the perimysium (Fig. 6). However, a few collagen fibres from the perimysium were confluent with the ORL (asterisks in Fig. 5).

As a declination of the preorbicularis fibres increased anteriorly, their orientation appeared approximately parallel to the skin (row D in Fig. 5). A dermal anchorage of the ORL was shown as a confluence of its fibroelastic tissue into the dermis.

## Discussion

**Figure 7:**
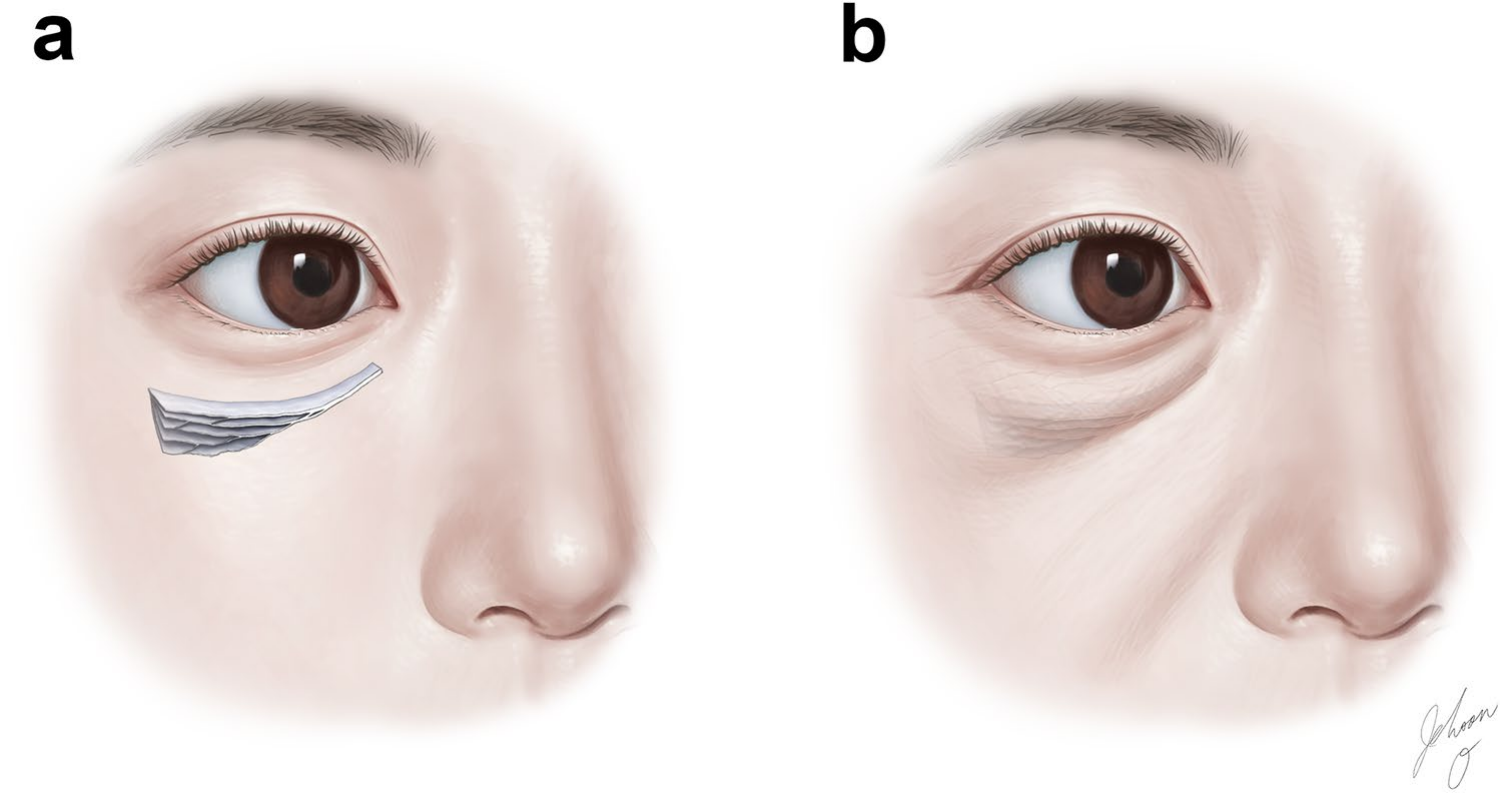
A model of the ORL appearing like puff pastry. The ORL appears in three dimensions as a multilayered meshwork of thin continuous fibroelastic plates (**a**), and its dermal attachment corresponds to a cutaneous groove or sagging bag (**b**).

The ORL has been described as originating from the lower orbital rim and traversing the OOc in a multilamellar fashion^2,3,5,7,18^. The present study has provided detailed mCT and histological findings indicating that the corresponding continuous ligamentous tissues typically appear to originate from the periosteum below the lower orbital rim. The VG and IF imaging verified that the ORL is a fibroelastic tissue with a histological continuity from its periosteal origination to dermal attachment. Like previous studies, the present mCT and histological investigations showed that the ORL exhibits discrete filamentous layers in the sagittal section^2,3,5,19^. However, the multidirectional observations performed using mCT demonstrated that overall the ORL comprises a multilayered meshwork of very thin continuous fibroelastic plates. Collectively, its 3D morphology appears like the inside of puff pastry, showing plexiform plates (Fig. 7a).

The number of retro-orbicularis fibres varied in this study. Previous studies found that the ORL comprised a single layer at the medial portion and bifurcated near the midpupillary line^7^. The ORL at the lateral portion appeared in two layers separated by 4–8 mm. This macroscopic appearance might represent a morphological tendency, in which the number, extent, structural complexity and ambiguity of the ORL layers increased in a lateral direction. However, it is rather difficult to definitively determine whether the ORL comprises one or two layers. Especially in the lateral area, increased layering with an appearance like that of puff pastry and structural ambiguity result in vertical widening of the ORL. The previously reported bilayered structure would have a macroscopic appearance of a lateral wide portion appearing like puff pastry with its upper and lower borders. The extent of the ORL and its number of layers also varied between individuals. In summary, the locations of these ORL layers and their borders might be more ambiguous than the descriptions in previous reports.

In 4 cases of 11 (36.4%), the uppermost fibre of the ORL seemed to originate from a very proximal part of the orbital septum or a junctional area between the periosteum and the orbital septum (Fig. 4d). Continuity of the uppermost fibre of the ORL with the periosteum was also clearly shown histologically in these cases. Thus, although the ORL could be macroscopically seen to begin with the orbital septum at its uppermost origin, the orbital septum would trivially affect any movements of the ORL. The retro-orbicularis fibres mostly separately traversed the muscle fascicles of the OOc. A few perimysia joined the outside portion of the ORL fibres. In immunohistochemical staining, perimysium did not contain elastin unlike ORL. In this regard, ORL and perimysium are not extended or derived from each other, they are distinct structures not only morphology but also constituents (Fig. 6). The direct bundles of the ORL—proceeding from the periosteum to the dermis as a single continuous ligament—would provide a direct anchorage to support both the OOc and the skin. Clinically, the complete resection of the retro-orbicularis fibres could eliminate any periosteal support of the OOc and its overlying dermis. Meanwhile, a cutaneous structure retaining the OOc could be sustained by the preorbicularis fibres with the perimysial retention. Attachment of the OOc to the dermis has been considered to contribute a lateral part of the hollow between the upper cheek and lower eyelid^1,2,20,21^. Accordingly, the palpebral portion of the OOc was tightly attached to the dermis and its lower border nearly corresponded to a cutaneous groove. The upper preorbicularis fibres also attached slightly below the lower border of the OOc, but the bundles of the ORL did not join the muscle fibres.

A tree model of arborized filamentous was previously suggested for delineating the ORL^22^. The present study also observed a tree appearance in the sagittal section, as a progressive divergence of the fibres. A particularly interesting observation was that the lower preorbicularis fibres reached not only the orbicularis region but also the prezygomatic region across the lower border of the OOc (Fig. 4b–d). These fibres corresponded to the zygomatic cutaneous ligament that was previously considered to be a distinct facial retaining ligament that is separate from the ORL. These two cutaneous ligaments might actually be two parts of a broadly extended ligamentous complex. The puff-pastry-like structure of the ORL has a distinct upper border and an ambiguous lower border. A clear upper border of the preorbicularis fibres could manifest as a visible cutaneous groove resulting from an accumulation of adipose tissue. Such a groove could be a tear trough or nasojugal groove medially and a palpebromalar groove laterally (Fig. 7b)^6,7^. In not only the lower orbicularis region but also the prezygomatic region, the skin and its underlying subcutaneous tissue were retained by the preorbicularis fibres. Shrinkage of the subcutaneous adipose tissue with aging could result in a sagging appearance in the prezygomatic region^21,23,24^. The sagging skin could be retracted upward by the preorbicularis fibres and the adipose tissue might accumulate within the lower prezygomatic region. This would be related to a cheek bulge previously delineated as the malar mound or festoons (Fig. 7b).

The dermal anchorage of the ORL could not be discriminated definitively as distinct fibres by pressing down perpendicularly on the skin surface. Instead, there was a fibroelastic combination of preorbicularis fibres with the dermis (row D in Fig. 5). Not only the ORL itself but also its dermal anchorage in a fibroelastic combination would be related to pathophysiological cutaneous laxation. Thus, histological alterations with aging, especially in elastic tissue, could induce sagging of the skin or produce a cutaneous groove.

The mCT observations with PTA tissue preparation enabled a detailed 3D examination of the ORL^13–17^. There are several contrast agents enhancing contrast of the soft tissue. Nierenberger *et al*. described the PTA’s contrast was superior to the Lugol solution^13^. Metscher reported the PTA is easier to handle and much less toxic than osmium, and he produced high-contrast x-ray images of a wide variety of soft tissues^14^. Furthermore, Nieminen *et al*. described the PTA-induced x-ray attenuation is a potential marker for non-destructive detection of articular cartilage collagen distributions in 3D^15^. As a non-destructive morphological method, PTA staining technique can avoid destruction of the original configuration of delicate retaining ligaments that can occur during manual dissection.

This study has verified that previous descriptions of the ORL are still largely accurate on the macroscopic scale, with disparities between the present and previous findings possibly attributable to the observational scale. The findings of this study suggest that the well-known cutaneous convexity or groove with aging in the suborbicularis and prezygomatic regions—such as the tear trough, palpebromalar groove or malar mound—should be reconsidered since it might be indiscriminately formed by a typical definite demarcation. Instead, these features appear to be a complicated outcome resulting from shrinkage of subcutaneous tissue, the accumulation of adipose tissue and retention of the ORL.

## Methods

Twenty-two specimens were obtained bilaterally from 11 non-embalmed human cadaver heads (Five females and six males, mean age 73.7 years, range 52–88). All cadavers utilized in this study were legally donated to the Surgical Anatomy Education Centre at Yonsei University College of Medicine. Specimens including periosteum, fat, muscle, subcutaneous tissue and skin were harvested from the inferior palpebral and infraorbital area, and immediately fixed with 10% formalin for 2~3 days. Eleven specimens were used for soft-tissue contrast staining to obtain mCT images, while the other 11 were used for histological staining. The specimens were demarcated into medial, central and lateral portions.

### Obtaining mCT images with PTA tissue preparation and three-dimensional image analysis

The specimens were serially dehydrated in a graded series of 30%, 50% and 70% ethanol solutions and then stained in 1% PTA solution with 70% ethanol for 5~7 days. The stained specimens were then scanned (Skyscan 1173, Bruker, Kontich, Belgium) at an image pixel size of 20 μm with 2240 × 2240 pixels. An electron accelerating voltage of 70 kV was used and a current was set to 114 μA. Detector timing was set to 500 ms and 0.3 degree rotation step was used. The scanned images were reconstructed using NRecon reconstruction software (Bruker). The data sets were imported into the CTVox software (Bruker) to crop a volume rendered in three dimensions. DicomCT (version 2.0, Bruker) was used to convert the data sets generated by the Skyscan system into DICOM format, and the DICOM data set was processed using Mimics (version 19, Materialise, Leuven, Belgium).

### Modified Verhoeff Van Gieson staining

The VG images were produced by first fixing specimens in paraffin and cutting them into 4-μm-thick sections before being mounted. The sections were then deparaffinized in xylene, followed by rehydration in a graded series of 100%, 95% and 70% ethanol solutions in serial washes for 3 minutes. After being immersed in distilled water for 5 minutes, the sections were placed in working Elastic Stain Solution (Abcam, Cambridge, UK) for 30 minutes and then washed in running tap water. They were differentiated using 2% ferric chloride, treated with 5% sodium thiosulphate for 1 minute, incubated in Van Gieson’s Solution for 3 minutes and then dehydrated rapidly using ethanol and cleared in xylene. The sections were imaged with the aid of a bright-field microscope (BX51, Olympus, Tokyo, Japan).

### IF procedure

The IF images were produced by immersing sections in a 0.01 M sodium citrate buffer solution (pH 6.0) and then boiling them in an autoclave at 120 °C for 10 minutes. After performing an antigen retrieval process, 1 mg/ml sodium borohydride solution in 0.1 M phosphate-buffered saline (pH 7.4) was applied for 10 minutes three times to remove autofluorescence. After blocking with 3% bovine serum albumin (BSA), the sections were incubated overnight at 4 °C with rabbit anticollagen type I antibody (1:200; Abcam) in 3% BSA. The sections were then incubated with goat antirabbit IgG Alexa Fluor 488 antibody (1:400; Abcam) for 2 hours at room temperature. Lastly, they were labelled for 2 hours with Phalloidin-iFluor™ 647 Conjugate (1:400; AAT Bioquest, Sunnyvale, CA). The sections were then mounted and stored in a dark slide box at 4 °C for 1~2 days. Images were taken with a WIBA (FITC) filter for collagen type I, a WIG (TRITC) filter for phalloidin-stained actin filaments and a WU (DAPI) filter for detecting autofluorescence of elastin^25^.

The ratio of the elastin volume to the collagen type I volume was quantitatively evaluated. We chose three slides of every eleven samples (a central portion) and then got an average value from high magnification images. After establishing the region of interests for each location, we measured the two-dimensional area of collagen type I signals and elastin signals at exactly same position using image J software (Java 1.8.0, National Institutes of Health, Bethesda, MD). And It was calculated as a ratio. The data were analyzed using SPSS Statistics (version 23, IBM Corporation, Armonk, NY).

## Electronic supplementary material

Supplementary Figures

Supplementary Video S1

## Data Availability

The datasets generated during and/or analysed during the current study are available from the corresponding author on reasonable request.

## References

[1] Schiller JD (2012). Lysis of the orbicularis retaining ligament and orbicularis oculi insertion: a powerful modality for lower eyelid and cheek rejuvenation. Plast Reconstr Surg..

[2] Muzaffar Arshad R., Mendelson Bryan C., Adams William P. (2002). Surgical Anatomy of the Ligamentous Attachments of the Lower Lid and Lateral Canthus. Plastic and Reconstructive Surgery.

[3] Kikkawa DO, Lemke BN, Dortzbach RK (1996). Relations of the superficial musculoaponeurotic system to the orbit and characterization of the orbitomalar ligament. Ophthalmic Plast Reconstr Surg..

[4] Wong CH, Mendelson B (2017). Extended Transconjunctival Lower Eyelid Blepharoplasty with Release of the Tear Trough Ligament and Fat Redistribution. Plast Reconstr Surg..

[5] Pessa JE, Garza JR (1997). The malar septum: the anatomic basis of malar mounds and malar edema. Aesthet Surg J..

[6] Hashem AM, Couto RA, Waltzman JT, Drake RL, Zins JE (2017). Evidence-Based Medicine: A Graded Approach to Lower Lid Blepharoplasty. Plast Reconstr Surg..

[7] Wong CH, Hsieh MK, Mendelson B (2012). The tear trough ligament: anatomical basis for the tear trough deformity. Plast Reconstr Surg..

[8] Wong CH, Mendelson B (2013). Facial soft-tissue spaces and retaining ligaments of the midcheek: defining the premaxillary space. Plast Reconstr Surg..

[9] Yang C, Zhang P, Xing X (2013). Tear trough and palpebromalar groove in young versus elderly adults: a sectional anatomy study. Plast Reconstr Surg..

[10] Alghoul M, Codner MA (2013). Retaining ligaments of the face: review of anatomy and clinical applications. Aesthet Surg J..

[11] Chan NJ (2018). Orbicularis retaining ligament release in lower blepharoplasty: assessing efficacy and complications. Ophthalmic Plast Reconstr Surg..

[12] Jacono AA, Malone MH (2017). Extended submuscular blepharoplasty with orbitomalar ligament release and orbital fat repositioning. JAMA Facial Plast Surg..

[13] Nierenberger M, Remond Y, Ahzi S, Choquet P (2015). Assessing the three-dimensional collagen network in soft tissues using contrast agents and high resolution micro-CT: Application to porcine iliac veins. C R Biol..

[14] Metscher BD (2009). MicroCT for comparative morphology: simple staining methods allow high-contrast 3D imaging of diverse non-mineralized animal tissues. BMC Physiol..

[15] Nieminen HJ (2015). Determining collagen distribution in articular cartilage using contrast-enhanced micro-computed tomography. Osteoarthritis Cartilage..

[16] Dunmore-Buyze PJ (2014). Three-dimensional imaging of the mouse heart and vasculature using micro-CT and whole-body perfusion of iodine or phosphotungstic acid. Contrast Media Mol Imaging..

[17] Karhula SS (2017). Effects of Articular Cartilage Constituents on Phosphotungstic Acid Enhanced Micro-Computed Tomography. PLoS One..

[18] Mojallal A, Cotofana S (2017). Anatomy of lower eyelid and eyelid-cheek junction. Ann Chir Plast Esthet..

[19] Hwang K, Nam YS, Kim DJ, Han SH (2008). Surgical anatomy of retaining ligaments in the periorbital area. J Craniofac Surg..

[20] Ghavami A (2008). The orbicularis retaining ligament of the medial orbit: closing the circle. Plast Reconstr Surg..

[21] Haddock Nicholas T., Saadeh Pierre B., Boutros Sean, Thorne Charles H. (2009). The Tear Trough and Lid/Cheek Junction: Anatomy and Implications for Surgical Correction. Plastic and Reconstructive Surgery.

[22] Kim, H. J., Seo, K. K., Lee, H. K. & Kim, J. Clinical anatomy of the Face for Filler and Botulinum Toxin Injection (1st ed.) 21–23 (Springer, 2016).

[23] Lucarelli MJ, Khwarg SI, Lemke BN, Kozel JS, Dortzbach RK (2000). The anatomy of midfacial ptosis. Ophthalmic Plast Reconstr Surg..

[24] Korn BS, Kikkawa DO, Cohen SR (2010). Transcutaneous lower eyelid blepharoplasty with orbitomalar suspension: retrospective review of 212 consecutive cases. Plast Reconstr Surg..

[25] Daamen WF (2003). Preparation and evaluation of molecularly-defined collagen-elastin-glycosaminoglycan scaffolds for tissue engineering. Biomaterials..

