# Supplementary material for: Three-dimensional structure of the orbicularis retaining ligament: an anatomical study using micro-computed tomography
Source: Sci Rep. 2018 Nov 19;8:17042. doi: 10.1038/s41598-018-35425-0 (PMC6242969; doi:10.1038/s41598-018-35425-0)
Supplement: Supplementary file 1 — Supplementary Figures [file 41598_2018_35425_MOESM1_ESM.pdf]

# Three-dimensional structure of the orbicularis retaining ligament: an anatomical study using micro-computed tomography


Jehoon O[#], Hyun-Jin Kwon[#], You-Jin Choi, Tae-Hyeon Cho and Hun-Mu Yang

**[#]co-1st authors. J.O. and H.J.K. contributed equally to the manuscript.**

**Department of Anatomy, Yonsei University College of Medicine, Seoul, Republic of Korea**

**Correspondence to:**

**Hun-Mu Yang, DDS, PhD**

Department of Anatomy, Yonsei University College of Medicine

Address: 50-1 Yonsei-ro, Seodaemun-gu, Seoul, 03722, Republic of Korea

Telephone: +82-2-2228-1649

E-mail: yanghm@yuhs.ac

**Authors information**


1. Jehoon O, MFA

Department of Anatomy, Yonsei University College of Medicine

Address: 50-1 Yonsei-ro, Seodaemun-gu, Seoul 03722, South Korea

E-mail: jhoon81@yuhs.ac

2. Hyun-Jin Kwon, PhD Student

Department of Anatomy, Yonsei University College of Medicine

Address: 50-1 Yonsei-ro, Seodaemun-gu, Seoul 03722, South Korea

E-mail: hjkwon128@yuhs.ac

3. You-Jin Choi, PhD

Department of Anatomy, Yonsei University College of Medicine

Address: 50-1 Yonsei-ro, Seodaemun-gu, Seoul 03722, South Korea

E-mail: CYJ7797@yuhs.ac

4. Tae-Hyeon Cho, BS

Department of Anatomy, Yonsei University College of Medicine

Address: 50-1 Yonsei-ro, Seodaemun-gu, Seoul 03722, South Korea

E-mail: CHOTH@yuhs.ac

# Supplementary Video S1

**Three-dimensional structure of the orbicularis retaining ligament**
Video showing the three-dimensional structure of the orbicularis retaining ligament (ORL) reconstructed from micro-computed tomography images.

# Supplementary Figure S1

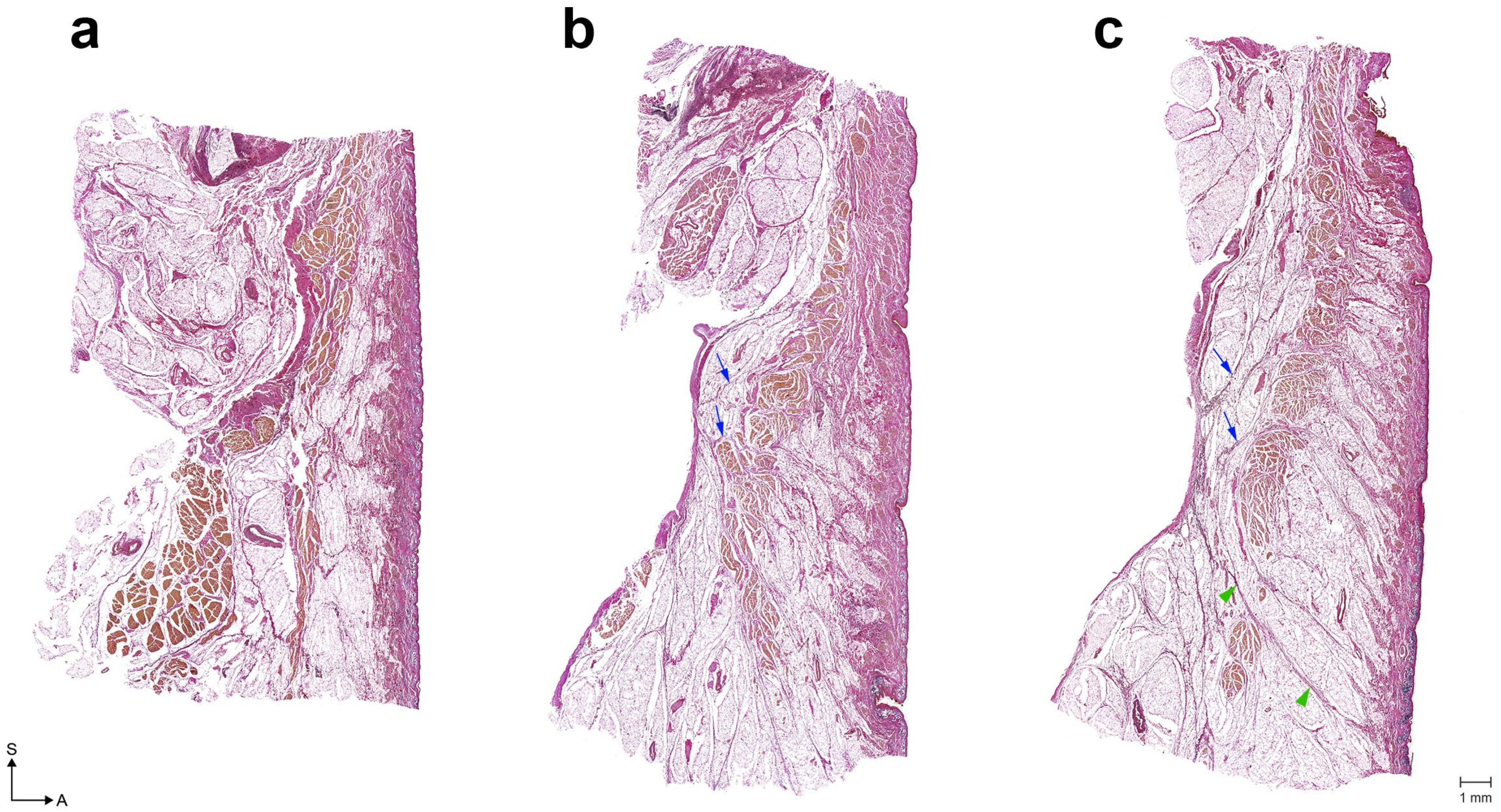


**Modified Verhoeff Van Gieson staining images**
Modified Verhoeff Van Gieson staining images of the medial (a), central (b) and lateral (c) portions of the ORL. S, sagittal; A, anterior.

# Supplementary Figure S2

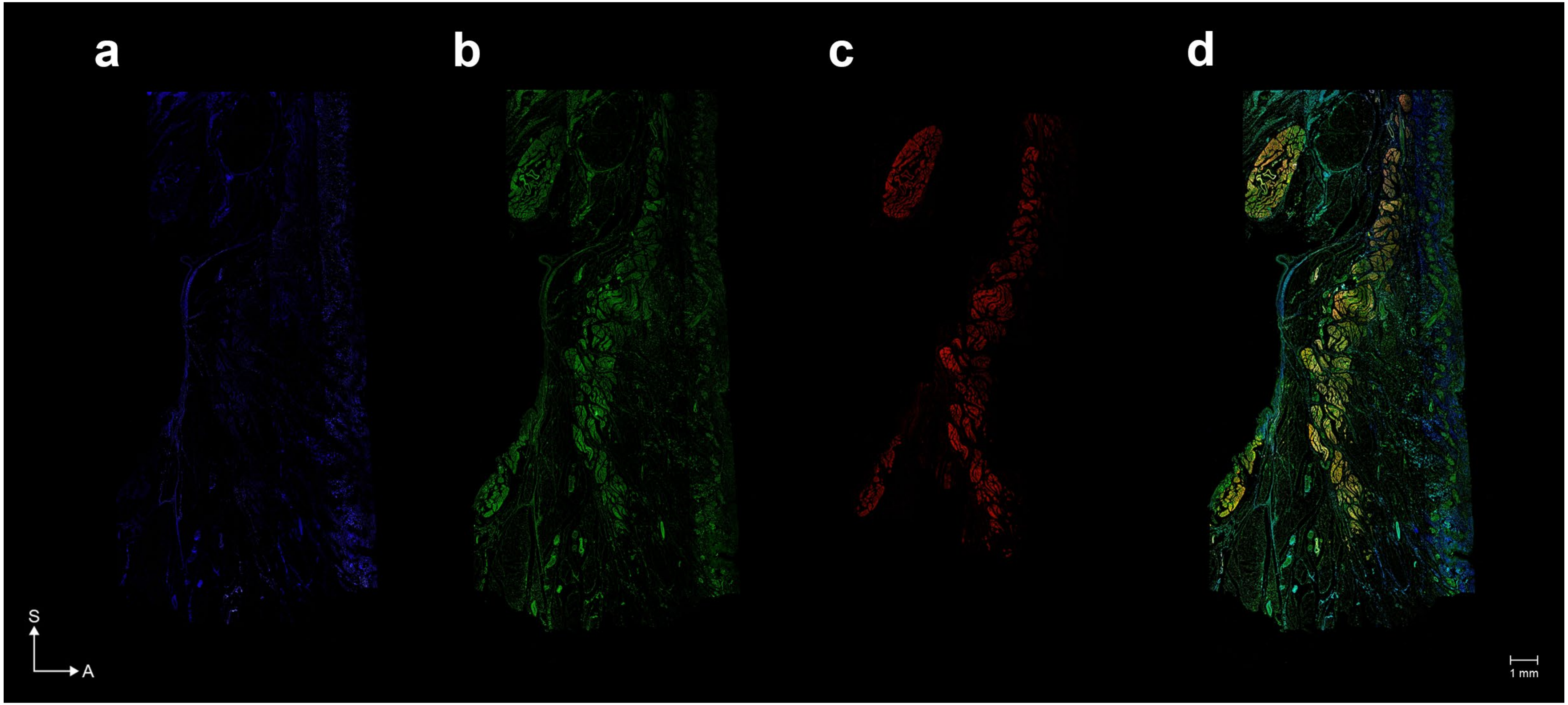


**Immunofluorescence of the ORL**
Immunofluorescence of the ORL for elastin (a, blue), collagen type I (b, green) and actin (c, red). (d) Merged image.
